# Reversed phase HPLC analysis of mobocertinib and its impurities and studies on the structure and biological activity of a new degradation product

**DOI:** 10.3389/fchem.2025.1659507

**Published:** 2025-09-22

**Authors:** Ronghua Ni, Jisu Qin, Wenyi Wu, Jinqiu Xu, Qunfeng Luo, Liangliang Cai

**Affiliations:** 1 Department of Pharmacy, Affiliated Hospital of Nantong University, Pharmacy School of Nantong University, Nantong, China; 2 Department of quality inspection, Sinopharm holding Nantong Ltd, Nantong, China; 3 School of Basic Medical Sciences, Jiangxi Medical College, Nanchang University, Nanchang, China

**Keywords:** mobocertinib, impurity, RP-HPLC, novel product, anticancer effect

## Abstract

**Background:**

Mobocertinib, an epidermal growth factor receptor tyrosine kinase inhibitor, is prescribed for the treatment of non-small cell lung cancer characterized by epidermal growth factor receptor exon 20 insertion mutations. The presence of impurities generated during its synthesis or storage may compromise the drug’s efficacy and safety. Therefore, a comprehensive investigation of these impurities and the implementation of rigorous quality control measures are of paramount importance. However, robust analytical methods for the simultaneous and accurate detection of mobocertinib and its related impurities are currently lacking.

**Methods:**

This study developed a novel reversed-phase high-performance liquid chromatography method (RP-HPLC) for separating and analysing mobocertinib and its impurities. An Agilent 5HC-C18 column (4.6 mm × 250 mm, 5 μm) was used to separate Mobocertinib and its related substances. The mobile phase composition, gradient elution program, and ultraviolet detection wavelength were optimized. Additionally, a new product (imp-A) was found during the forced degradation test. Its structure was elucidated by RP-HPLC, nuclear magnetic resonance (NMR) and high resolution mass spectrometry (HRMS). The biological activity of imp-A was preliminarily evaluated by methyl thiazolyl tetrazolium (MTT) assay.

**Results:**

The RP-HPLC method developed in this study was validated in accordance with ICH guidelines, demonstrating satisfactory specificity, precision, stability, repeatability, accuracy, and robustness. The method exhibited good linearity over the concentration range of 0.1–20 μg mL-1. The limits of detection and quantitation for mobocertinib were determined to be 0.02 μg mL-1 and 0.05 μg mL-1, respectively. The structure of imp-A was successfully characterized, and its formation mechanism was elucidated. Furthermore, imp-A was found to inhibit the growth of various tumor cell lines.

**Conclusion:**

The developed RP-HPLC method is suitable for the simultaneous detection of mobocertinib and its impurities, providing significant advantages for process development and quality control. Imp-A, a novel compound, demonstrated promising anticancer activity in vitro. However, further in vivo studies are required to fully assess its therapeutic potential, which may hold promise for clinical applications in cancer treatment.

## 1 Introduction

Targeted treatment is an important method for treating non-small cell lung cancer (NSCLC), especially for patients with target mutations. Epidermal growth factor receptor (EGFR)-activating mutations are frequent and primarily involve exons 18 to 21 of the EGFR tyrosine kinase domain (Sharma et al., 2007). In-frame deletions in exon 19 and L858R substitution in exon 21 are common EGFR-activating mutations (Kobayashi and Mitsudomi, 2016). The T790M is one of the reasons for drug resistance (Yu et al., 2013). In recent years, EGFR exon 20 insertion (EGFRex20ins) has received considerable attention. This insertion is associated with poor prognosis and can cause conformational changes to the receptor, which results in steric hindrance and prevents binding to the active site (Vyse and Huang, 2019). As a result, EGFR ex20ins is also associated with *de novo* resistance to first- and second-generation EGFR tyrosine kinase inhibitors (TKIs) (Gonzalvez et al., 2021; Vyse and Huang, 2019). EGFRex20ins mutations constitute 6%–12% of EGFR kinase domain mutations (Arcila et al., 2013; Kobayashi and Mitsudomi, 2016; Oxnard et al., 2013; Riess et al., 2018). Hence, EGFRex20ins are important targets for NSCLC treatment.

Mobocertinib (TAK788) is a first-in-class EGFR TKI that is selective for EGFRex20ins. Mobocertinib belongs to the third generation EGFR-TKI as osimertinib (Yuan et al., 2022). The use of mobocertinib has received considerable attention. The efficacy of mobocertinib has been studied *in vitro* and *in vivo*, and satisfactory anticancer activity of mobocertinib against EGFRex20ins mutations has been demonstrated (Gonzalvez et al., 2021). The two active metabolites of mobocertinib (AP32960 and AP32914) have a potency equal to that of the parent drug. In a Phase 1/2 study, mobocertinib induced durable responses in adults with previously treated EGFR ex20ins + metastatic NSCLC (Garcia Campelo et al., 2022). Recently, some studies have focused on bioavailability, pharmacokinetics, metabolism and excretion of mobocertinib in rats, healthy participants and patients with NSCLC (Chen et al., 2024; Gupta et al., 2022; Hanley et al., 2024; Li et al., 2021a). In addition, Gunturu. Raviteja and Kantipudi Rambabu develop a high-performance liquid chromatography (HPLC) method for determination of mobocertinib in a pharmaceutical dosage form (Rambabu and Raviteja, 2021). It is worth noting that the biosynthesis and degradation of mobocertinib may produce impurities that have quite important safety and therapeutic repercussions and must be considered in process preparation studies and for quality control (Baksam et al., 2021; Gopireddy et al., 2021; Li et al., 2021b; Lu et al., 2019). However, studies about impurities of mobocertinib were rare. Shweta Mishra et al. used LC-MS/MS and NMR to isolate, identify and characterize structure of forced degradation products of mobocertinib (Mishra et al., 2024). Whereas, they could not detect the mobocetinib and its impurities simultaneously in these studies. Thus, it is crucial to develop a method for detecting mobocertinib and its impurities at the same time.

Reversed-phase HPLC (RP-HPLC) is an ideal technique for detecting drugs and their impurities and has been applied to different kinds of drugs (Li et al., 2020; Tome et al., 2020; Wang et al., 2020). The advantages of RP-HPLC include convenience, simplicity, stability, and low cost. Consequently, we developed an RP-HPLC method to detect mobocertinib and its impurities, which is reported here for the first time. The proposed method was validated according to International Conference Harmonization (ICH) guidelines (ICH, 2023). Specifically, mobocertinib and its impurities (imp-A–F) were completely separated. The system suitability and specificity, precision, stability and robustness of the method were subsequently investigated. The lowest limit of detection (LOD), lowest limit of quantitation (LOQ), linearity and recovery of the RP-HPLC method were determined. Notably, a novel substance was discovered in this study. The structure of the substance was resolved successfully through nuclear magnetic resonance (NMR) and high resolution mass spectrometry (HRMS), and the anticancer effect of the substance was evaluated. It is our hope that this novel method will provide an effective way to detect mobocertinib and its impurities and that the newly discovered substance will provide additional treatment options for cancer patients.

## 2 Materials and methods

### 2.1 Chemicals and reagents

Mobocertinib (purity 99.3%) was obtained from Ji Nan Shandong Shenlikanghua Co., Ltd., and acetonitrile (ACN) and methanol (MeOH) (HPLC grade) were obtained from Merck KGaA. Process-related impurities (imp-B, C, D, E and F) were obtained from Haian Aila Co., Ltd. Their purities are 96.7%, 99.2%, 98.6%, 99.3% and 99.5%, respectively. The basic hydrolysis product (imp-A) was prepared in our laboratory and its impurities is 97.8%. Hydrochloric acid (HCl), phosphoric acid, sodium hydroxide (NaOH), hydrogen peroxide (H_2_O_2_) and triethylamine (analytical grade) were purchased from China National Pharmaceutical Group Corporation. Dulbecco’s modified Eagle’s medium (DMEM), Roswell Park Memorial Institute-1640 (RPMI-1640), foetal bovine serum (FBS), phosphate buffer solution (PBS), penicillin and streptomycin were purchased from Gibco, Inc. 3-(4,5-Dimethyl-2-thiazolyl)-2,5-diphenyl-2-H-tetrazolium bromide was obtained from Beyotime Biotechnology.

### 2.2 Instruments

This study was performed using an RP-HPLC instrument (Agilent 1,200, Agilent, United States) equipped with an ultraviolet–visible (UV–vis) spectrometer (Cary 300, Varian, United States) and a Milli-Q water purification system. An RP-HPLC instrument (Shimadzu 20AD, Shimadzu, Japan), NMR instrument (Bruker 600 M, Bruker, Germany) and HRMS (Agilent 6,540, Agilent, United States) were also utilized. In addition, a preparative column (Eclipse XDB-C18, Agilent, United States), a microplate reader (iMark, Bio-Rad, United States), a pH meter (PHS-3C, Leici, China) were used.

### 2.3 HPLC conditions

Mobocertinib and its related impurities were separated and detected using an Agilent 5HC-C18 column (4.6 mm × 250 mm, 5 μm). The mobile phase was a blend of an aqueous solution and ACN (9:1, v:v). The aqueous solution consisted of a 2 mM potassium dihydrogen phosphate (KH_2_PO_4_) solution and 0.4% triethylamine. The pH of the aqueous solution was adjusted to 2.5 by adding phosphoric acid. ACN was used as the mobile phase B. Gradient elution was carried out as follows: 0–2 min, at 10% solvent B; 2–8 min, from 10% to 30% solvent B; 8–25 min, from 30% to 40% solvent B; 25–40 min, from 40% to 90% solvent B; 40–45 min, at 90% solvent B; 45–46 min, from 90% to 10% solvent B; and 46–55 min, at 10% solvent B. The wavelength for UV detection was set at 330 nm. The flow rate was 1.0 mL min^-1^. The sample injection volume was 10 µL.

### 2.4 Preparation, purification and structural confirmation of imp-A

To prepare imp-A, 300 mg of mobocertinib was weighed and placed into a 500 mL round-bottom flask, followed by the addition of 150 mL of MeOH. After mobocertinib was dissolved completely, 75 mL of 1 mM NaOH was added, and the mixture was stirred at 80 °C overnight. Then, 1 mM HCl was added until the pH of the solution reached approximately 7.0. Afterwards, the MeOH in solution was removed by rotary evaporation. Finally, the residue was removed by freeze-drying, and the crude products were obtained.

The crude products were purified through semipreparative LC. Specifically, a preparative column (Agilent Eclipse XDB-C18, 250 × 9.4 mm, 5 μm) was used to purify the crude products of imp-A. The mobile phase consisted of water and MeOH (4:6, v:v), and the elution time was 8 min. The flow rate was 4 mL min^-1^. The eluent was collected. After rotary evaporation and freeze-drying, purified imp-A was obtained, and the purity was determined via HPLC according to the HPLC conditions described in Section 2.3.

The structure of imp-A was determined by NMR (^1^H NMR, ^13^C NMR and 2D NMR) and high-resolution MS analyses. Specifically, 10 mg of imp-A or 20 mg of imp-A was dissolved in DMSO-d6 for ^1^H NMR, ^13^C NMR and 2D NMR detection. For MS detection, imp-A was dissolved in MeOH at a concentration of 1 μg mL^-1^.

### 2.5 Preparation of stock solution

#### 2.5.1 Standard stock solution of mobocertinib

Ten milligrams of mobocertinib was accurately weighed and dissolved in MeOH/H_2_O (50:50, v:v). Then, the sample was transferred to a 10 mL volumetric flask and diluted to volume to prepare a standard solution (1 mg mL^-1^).

#### 2.5.2 Standard stock solutions of mobocertinib-related impurities

The impurities in mobocertinib (imp-A, B, C, D, E and F) were accurately weighed and diluted to a concentration of 1 mg mL^-1^, as described above, to prepare standard stock solutions.

### 2.6 Preparation of a system suitability solution

To prepare a system suitability solution, an acid degradation solution and an oxidative degradation solution were mixed in equal volumes. The following steps were used to prepare the acid degradation solution. First, approximately 10 mg of mobocertinib were placed in a 10 mL volumetric flask and dissolved in 5 mL of MeOH to a concentration of 2 mg mL^-1^. Second, 2.5 mL of 1 M HCl were added to the flask, and the resulting mixture was incubated for 6 h at 80 °C. Next, 1 M NaOH was added to the mixture to adjust the pH to neutral. Finally, the solution was diluted to volume with a MeOH/H_2_O (50:50, v:v) solvent. The oxidative solution was prepared by dissolving 10 mg of mobocertinib in MeOH to obtain a 2 mg mL^-1^ solution as described above, adding 5 mL of 30% H_2_O_2_, and diluting the mixture to volume with a solvent. The reaction was terminated after 22 h by the addition of manganese dioxide (MnO_2_). The solution was collected by removing the MnO_2_ and diluting the sample to the desired volume with a solvent.

### 2.7 Preparation of sample solution

The sample solution was prepared by accurately weighing approximately 10 mg of mobocertinib, which was then dissolved in a mixture of MeOH and H_2_O (50:50, v:v) to a concentration of approximately 1 mg mL^-1^.

### 2.8 Investigation of anticancer activities *in vitro*

The anticancer activities of imp-A were evaluated *in vitro*. A549, MDA-MB-231, HepaRG, PANC-1 and MKN-1 cells were use in this study. The HepaRG cell line was acquired from Shanghai Guan Dao Biological Engineering Co., Ltd. (Shanghai, China). A549, PANC-1, and MDA-MB-231 cells were sourced from the Cell Bank of the Chinese Academy of Sciences (Shanghai, China). The MKN-1 cell line was provided by the Laboratory of Clinical Medicine Center, Affiliated Hospital of Nantong University. The five kinds of tumour cells were cultured in 96-well plates for 24 h. Imp-A (7.5, 15, 30, 60 and 120 μg mL^-1^) were added to the wells at various concentrations, respectively. The imp-A solution were removed after 48 h, and 100 μL of MTT (1 mg mL^-1^) were incubated with the cells for 4 h at 37 °C. Finally, 150 μL of DMSO were added to the cells, and the resulting mixture was incubated for an additional 30 min at 37 °C. The absorbance was measured at 490 nm by a microplate reader.

## 3 Results and discussion

### 3.1 Method development

**FIGURE 1:**
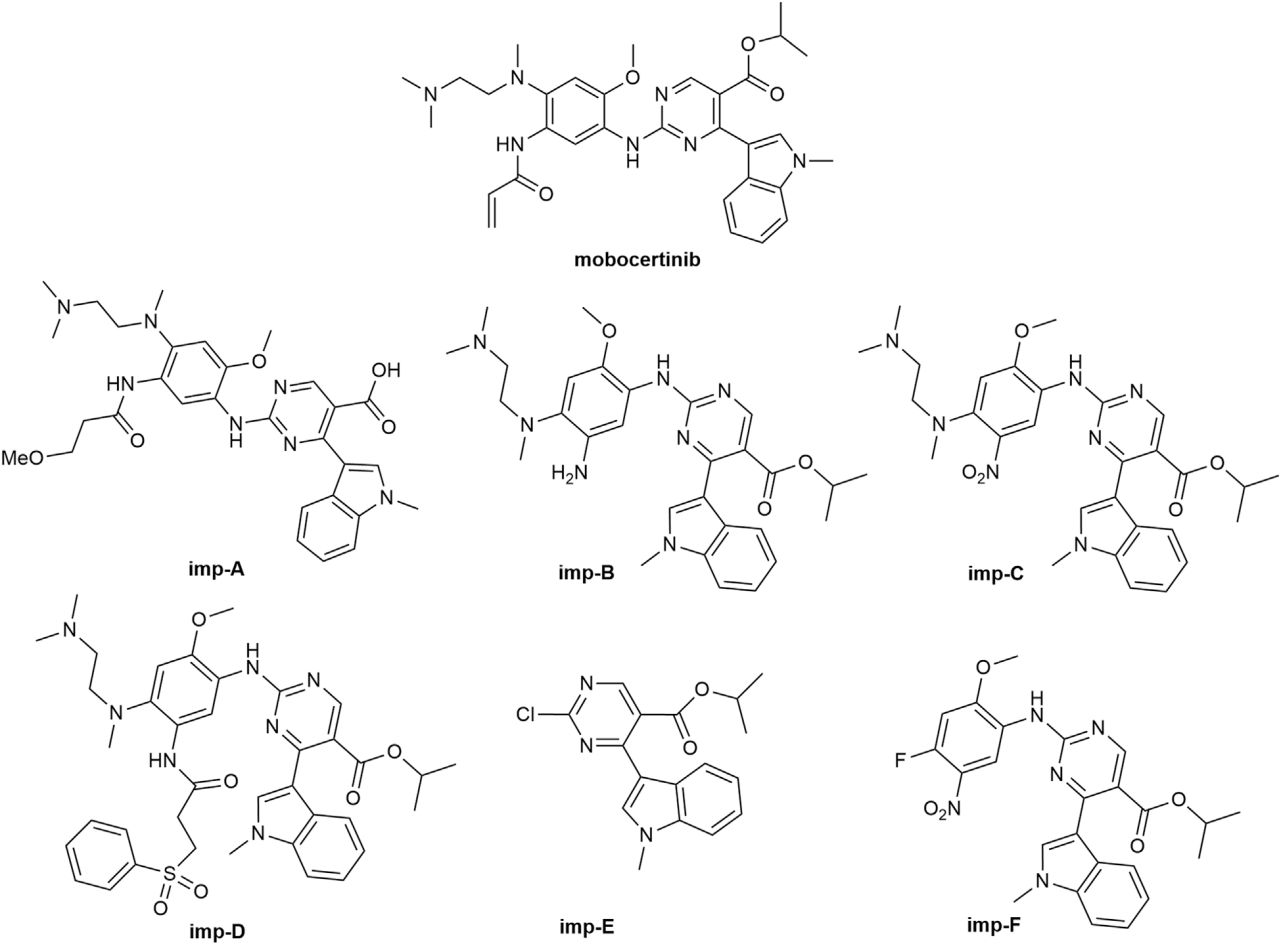
The structures of the mobocertinib and its impurities.

Mobocertinib is a small-molecule inhibitor of the EGFRex20ins mutation. Impurities, such as raw materials and intermediate products, may be introduced during synthesis. Degradation-related substances are also important sources of impurities. A preliminary experiment revealed six kinds of primary impurities of mobocertinib. Imp-A is a novel product of basic hydrolysis. Imp-B-F are all intermediate products. The structures of mobocertinib and its impurities are shown in Figure 1. In this study, an RP-HPLC method was developed for detecting mobocertinib and its impurities. The solvent and HPLC conditions used for the method were optimized. Considering solubility, stability, and cost, MeOH/H_2_O (v:v, 1:1) was eventually selected as the solvent. The results of the optimization of the detection wavelength and HPLC conditions are presented in Supplementary Figures S1, S2, respectively.

### 3.2 Method validation

The HPLC method for the detection of mobocertinib and its impurities was successfully validated according to ICH guidelines (ICH, 2023). The validation parameters included the specificity, linearity, accuracy, precision, LOD, LOQ, stability and robustness

#### 3.2.1 Specificity

**FIGURE 2:**
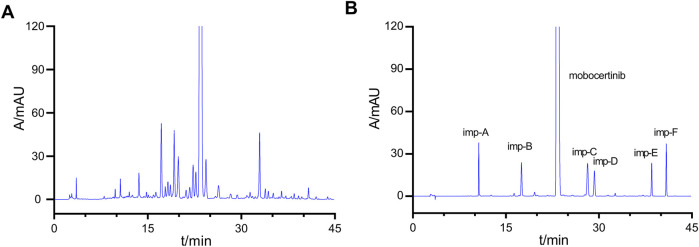
Chromatograms of the system suitability solution **(A)** and the solution of mixed impurities **(B)**.

Good specificity is a precondition for the development of an RP-HPLC method. Here, a system suitability solution was prepared with acid and oxidative degradation products and detected by HPLC. The HPLC conditions have been described previously. The chromatogram of the system suitability solution is presented in Figure 2A. The resolutions of the mobocertinib peak and the two adjacent peaks were 1.68 and 2.48, respectively, both of which were greater than 1.5. The minimal resolution was 1.81 for the adjacent known impurity peaks and 1.42 between the peaks of the two unknown impurities. These results indicate satisfactory system suitability.

A mixed solution was prepared to evaluate the specificity of the method. First, 10 mg of mobocertinib were weighed into a 10 mL volumetric flask. Then, 0.1 mL of a stock solution of imp-A–F was added to the prepared mobocertinib solution and diluted to volume with MeOH/H_2_O (50:50, v:v). Finally, the sample was tested under HPLC conditions. The results are presented in Figure 2B. The retention time of imp-A-F were 10.629, 17.520, 28.176, 29.282, 38.514, 40.874 min, respectively.

#### 3.2.2 Forced degradation test

**FIGURE 3:**
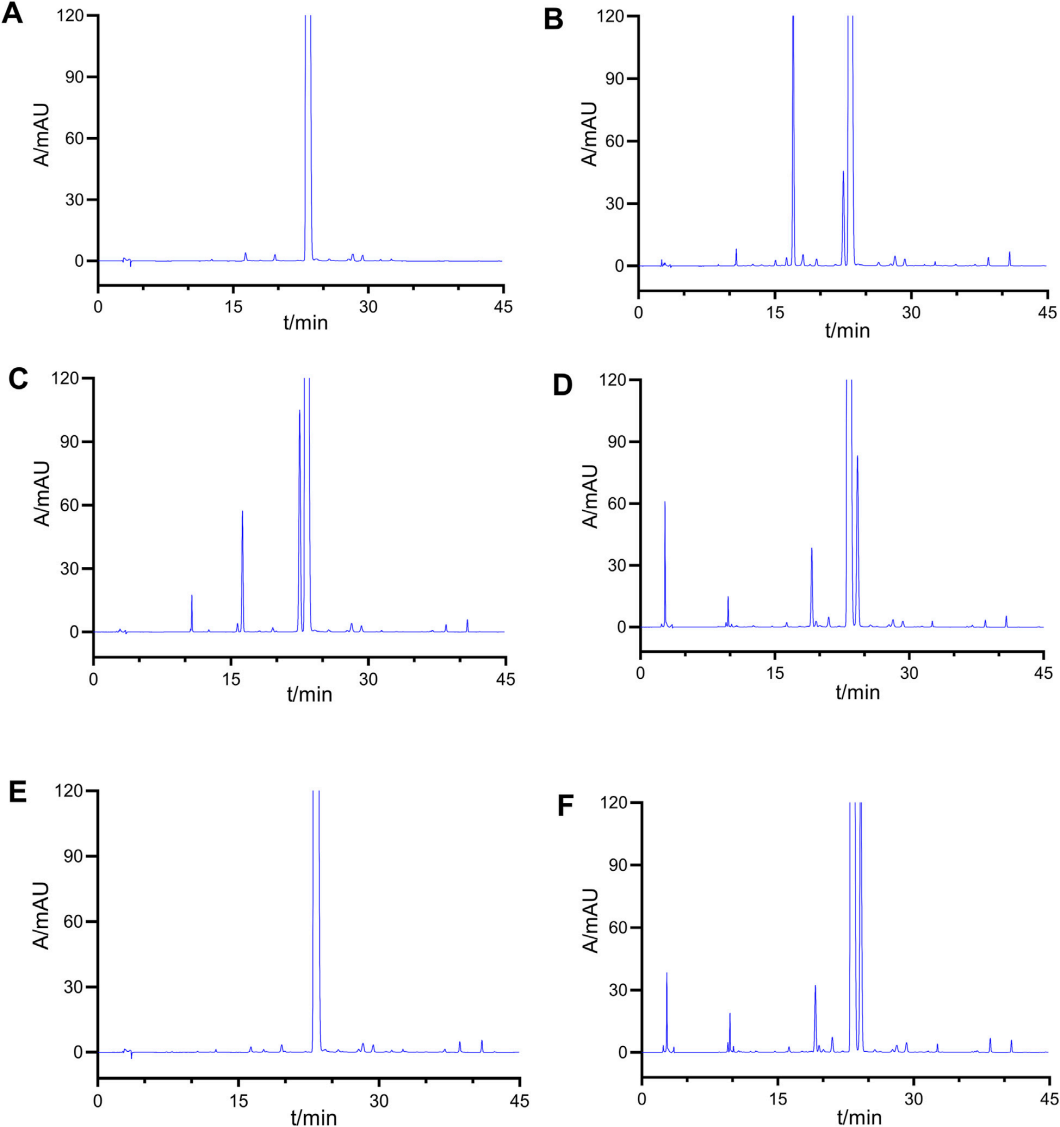
HPLC chromatograms of mobocertinib samples: non-degraded **(A)**, acid-degraded **(B)**, base-degraded **(C)**, oxidatively degraded **(D)**, heat-degraded **(E)**, and photolytically degraded **(F)**.

The forced degradation test was carried out by dissolving approximately 10 mg of mobocertinib in MeOH and subjecting the solution to different stress conditions, including acid and base hydrolysis, oxidation, photolysis, and heat stress. Specifically, the mobocertinib solution was treated with 1 mol mL^-1^ HCl for 4 h at 80 °C and 1 mol mL^-1^ NaOH for 1 h at room temperature (RT) for acid and base hydrolysis, respectively. The mobocertinib solution was subjected to oxidation by being incubated for 12 h at RT. Heat-stress tests were performed by increasing the temperature of the mobocertinib solution to 80 °C for 7 days. Photolytic stress was applied via an LED tube (4,500 lx) for 16 days. The samples were then detected under the conditions described in Section 2.3. The chromatograms are presented in Figure 3, and the results are shown in Supplementary Table S1. Under the different stress conditions, the minimal resolutions between the mobocertinib peak and all the impurity peaks were >1.5, and the resolutions of all the impurity peaks were >1.2, meeting the resolution requirement. Equilibrium occurred from 95.3% to 104.2%, which is considered a balanced range.

#### 3.2.3 Linearity

The linearity of mobocertinib and its impurities was investigated in the concentration range of 0.1–20 μg mL^-1^. Stock solutions of mobocertinib and its impurities were diluted to concentrations of 0.1, 0.2, 0.5, 1, 2, 5, 10, and 20 μg mL^-1^ with MeOH/H_2_O (50:50, v:v). Next, 10 μL of each of the prepared solutions were injected and analysed under the HPLC conditions, as described previously. Finally, the linearity of the seven substances was calculated. The regression curves are shown in Supplementary Figure S3, and the standard calibration curves and correlation coefficients are presented in Table 1.

**TABLE 1:** Linearity, LOD and LOQ of mobocertinib and its impurities.

Substance	Standard calibration curves	Correlation coefficient	LOD (μg mL^-1^)	LOQ (μg mL^-1^)
mobocertinib	y = 28.623x+5.9467	0.9991	0.02	0.05
impA	y = 16.681x+2.7864	0.9997	0.05	0.13
impB	y = 19.266x+2.8877	0.9994	0.03	0.07
impC	y = 28.382x+6.0785	0.9994	0.03	0.08
impD	y = 17.851x+2.7824	0.9995	0.04	0.10
impE	y = 16.879x+1.8079	0.9997	0.04	0.11
impF	y = 21.775x+3.5244	0.9998	0.02	0.06

#### 3.2.4 LOD and LOQ

The LODs and LOQs of mobocertinib and imp-A–F were calculated to evaluate the detectability of the method. Standard stock solutions of mobocertinib and the impurities A–F were gradually diluted and detected under the HPLC conditions described in Section 2.3. Finally, the signal-to-noise (S/N) ratio was calculated. The LOD and LOQ were defined as the analyte concentrations at S/N ratios of 3:1 and 10:1, respectively. Table 1 shows the calculated LODs and LOQs of mobocertinib and imp-A–F after six injections.

#### 3.2.5 Solution stability

The stability of the sample solution was tested at different time points at RT. The stability was evaluated in terms of the indicators shown in Table 2. No discernible changes in the respective three indicators were observed when the sample solution was allowed to stand at RT for 24 h. These results demonstrated that the sample solution was stable within 24 h at RT.

**TABLE 2:** Test results of mobocertinib sample solution stability.

Time (h)	Number of impurities	Content of maximum single impurity (%)	Content of total impurities (%)
0	4	0.17	0.58
1	4	0.16	0.58
2	4	0.15	0.60
4	4	0.18	0.59
6	4	0.17	0.62
8	4	0.19	0.63
12	4	0.20	0.65
24	4	0.21	0.68

The stabilities of imp-A-F were also evaluated. Ten milligrams of mobocertinib were weighed in a 10 mL volumetric flask. Then, 0.2 mL of a stock solution of imp-A–F was added to the prepared mobocertinib solution and diluted to volume with MeOH/H_2_O (50:50, v:v). Finally, the mixed solution was detected at 0, 1, 2, 4, 6, 8, 12, and 24 h and the chromatograms were recorded. The RSDs for the peak areas of mobocertinib and imp-A-F were 0.78%, 0.86%, 0.69%, 1.35%, 1.71%, 0.92% and 1.48%, respectively.

#### 3.2.6 Precision

The precision of the method was determined by analysing the mixed solutions prepared for the stability study in sextuplicate and recording the retention times and peak areas of mobocertinib and imp-A-F. The RSDs of the retention times for mobocertinib and imp-A-F were 0.08%, 0.12%, 0.11%, 0.15%, 0.09%, 0.15%, and 0.07%, respectively. The RSDs of the peak areas for mobocertinib and imp-A-F were 0.68%, 1.25%, 0.93%, 1.34%, 1.02%, 1.13%, and 1.67%, respectively. All the RSDs were <2%, indicating good precision.

#### 3.2.7 Repeatability

The repeatability of the method was evaluated by preparing a mixed solution in sextuplicate and testing the samples under the HPLC conditions described in Section 2.3. The RSDs of imp-A-F were 1.51%, 1.62%, 1.73%, 1.82%, 1.35%, and 1.89%, respectively, indicating good repeatability.

#### 3.2.8 Recovery

To calculate the recovery of the method, three different levels (50%, 100%, 150%) were established, and a concentration of 2 μg mL^-1^ corresponded to 100%. The test solutions were prepared by accurately weighing 10 mg samples of imp-A-F, adding the samples to a 50 mL volumetric flask, and dissolving the samples in MeOH/H_2_O (50:50, v:v). Then, 1 mL of each sample solution was diluted to 10 mL to prepare a concentrated solution. Each sample solution was prepared in triplicate. A 2 mg mL^-1^ mobocertinib solution was prepared and spiked with a concentrated solution of imp-A-F. Next, 5 mL of a mixture of the mobocertinib solution (2 mg mL^-1^) and 1 mL of a concentrated solution (20 μg mL^-1^) of imp-A-F were transferred to a 10 mL-volumetric flask. The concentrations of mobocertinib and imp-A-F in the final solution were 1 mg mL^-1^ and 2 μg mL^-1^, respectively. Imp-A-F solutions were prepared at concentrations of 1 μg mL^-1^ and 3 μg mL^-1^ as described above. These sample solutions were subsequently analysed, and the results are shown in Supplementary Table S2. The lowest recoveries of imp-A-F were 93.97%, indicating good accuracy for the method.

#### 3.2.9 Robustness

The robustness of the method was investigated by adjusting the initial ratio of the mobile phases A and B and changing the wavelength, flow rate, and pH of the mobile phase. The specific conditions are listed in Supplementary Table S3.

Under the specified conditions, the minimum resolution was 1.61 between the mobocertinib peak and the adjacent impurity peaks and 1.39 (>1.2) between the other impurity peaks. There was no noticeable change in the number or content of impurities. Impurity detection was not affected by variations in the column temperature, wavelength, or pH of the mobile phase. Changes in the flow rate, initial proportion and chromatographic column of the mobile phase had little influence on the retention time or resolution. Fortunately, minor adjustments in these parameters did not noticeably affect the detection results, successfully demonstrating the robustness of the method.

### 3.3 Characterization of imp-A

**FIGURE 4:**
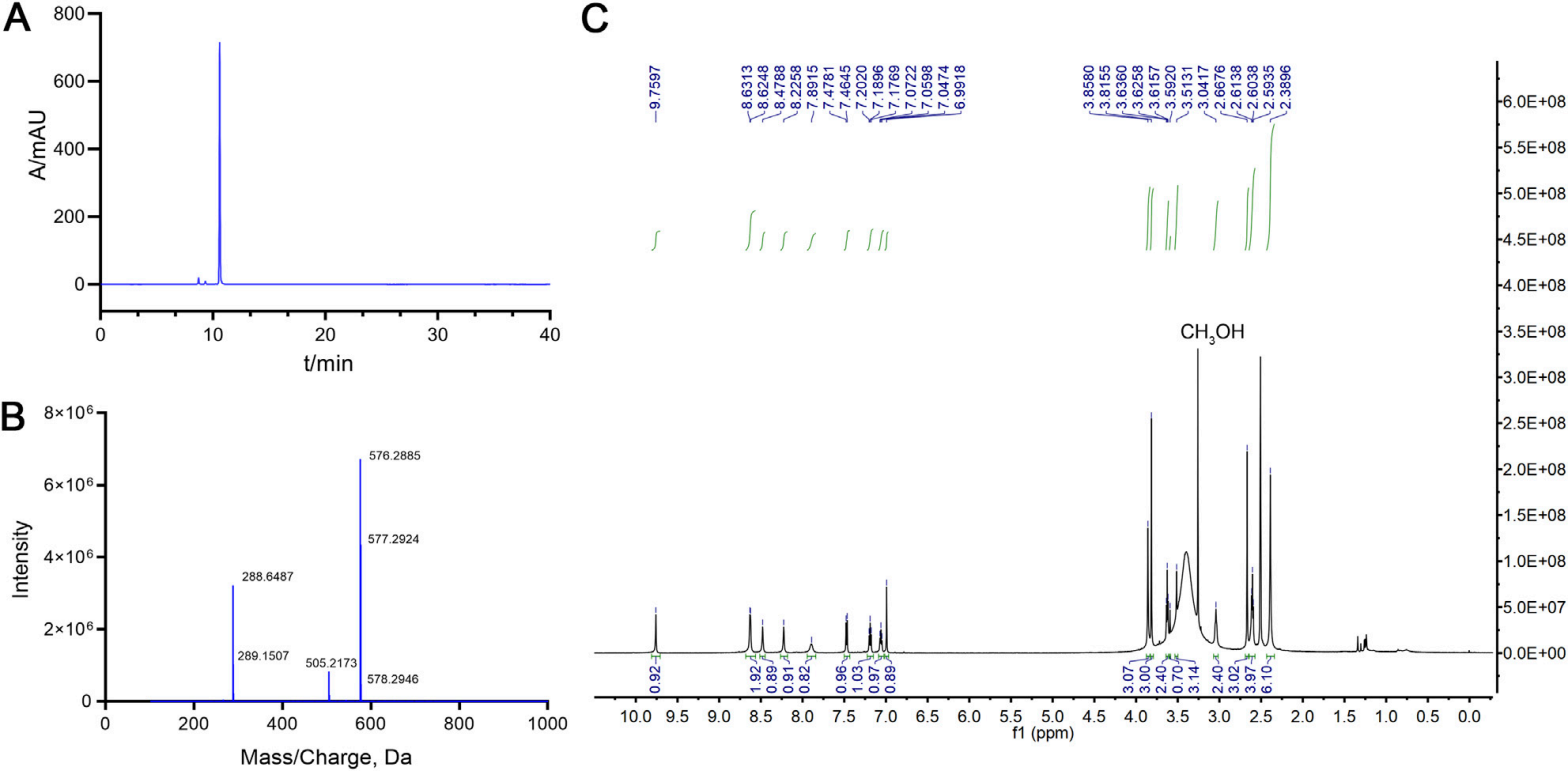
The spectra of imp-A: HPLC **(A)**, HRMS **(B)** and 1H NMR **(C)**. 1H NMR (600 MHz, DMSO) δ 9.76 (s, 1H), 8.63 (d, J = 3.9 Hz, 2H), 8.48 (s, 1H), 8.23 (s, 1H), 7.89 (s, 1H), 7.47 (d, J = 8.1 Hz, 1H), 7.19 (t, J = 7.5 Hz, 1H), 7.06 (t, J = 7.4 Hz, 1H), 6.99 (s, 1H), 3.86 (s, 3H), 3.82 (s, 3H), 3.63 (t, J = 6.1 Hz, 2H), 3.59 (s, 1H), 3.51 (s, 3H), 3.04 (s, 2H), 2.67 (s, 3H), 2.60 (t, J = 6.1 Hz, 4H), 2.39 (s, 6H).

**FIGURE 5:**
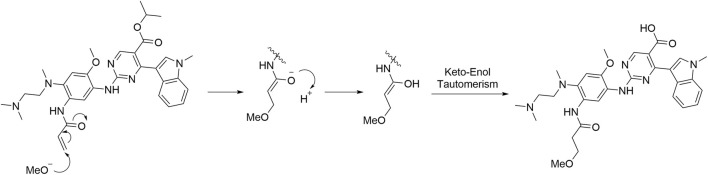
The formation mechanism of imp-A.

Imp-A was prepared in our laboratory according to the procedure described in Section 2.4. The corresponding chromatogram is presented in Figure 4A. The retention time was 10.74 min. The purity was calculated to be approximately 97.8%. Imp-A was characterized by NMR and HRMS. These techniques are used to identify and quantify the elemental composition of compounds (Wang et al., 2023). The HRMS was used to detect the molecular weight of imp-A, the spectrum is presented in Figure 4B. The molecular weight of imp-A is 575.2885 Da. NMR was used to characterize the structure of imp-A in detail. The ^1^H NMR spectrum of imp-A are shown in Figure 4C and the signals of H atoms of imp-A are indicated in Table 3. The ^13^C NMR spectra of imp-A are shown in Supplementary Figure S4. Supplementary Figures S5, S6 exhibit the full HMBC spectrum and locally amplified HMBC spectrum of imp-A, respectively. Through these results, we successfully inferred the structure of imp-A (Figure 1). In addition, the formation mechanism was revealed in Figure 5 which follows a Michael addition reaction mechanism. Since a Michael receptor, α, β-unsaturated amide exists in the structure of mobocertinib, it is prone to be attacked by electron rich group. Under the forced degradation condition (NaOH was used), MeO- formed and the double bond of the Michael receptor was attacked by MeO-. Then, the intermediate underwent keto-enol tautomerism to provide imp-A.

**TABLE 3:** ^1^H NMR assignment of imp-A.

δ	Number of H atom	Structure
9.76, 8.63, 8.48, 8.23, 7.89, 7.47, 7.19, 7.06, 6.99	H1–8, 10, 21	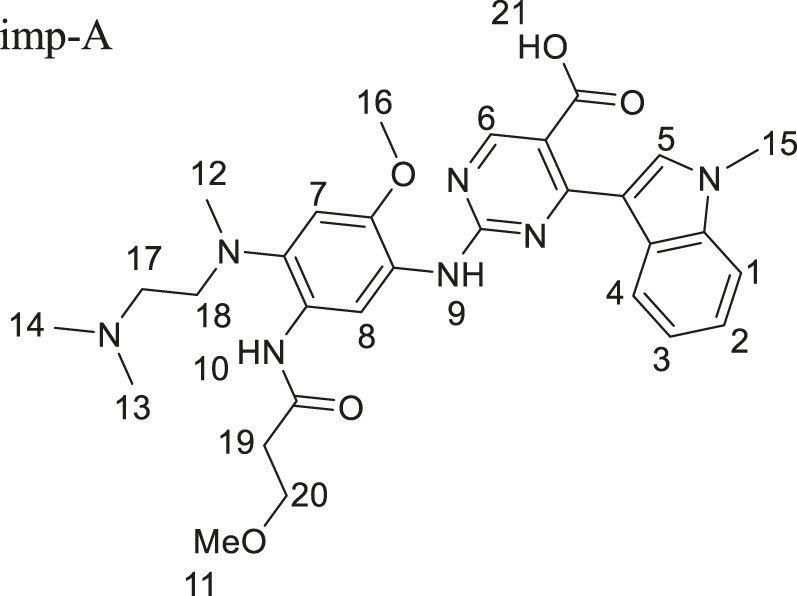 imp-A
3.86, 3.82	H 15, 16	
3.63	H 20	
3.59	H 9	
3.51	H 11	
3.04, 2.6	H 17, 18, 19	
2.67	H 12	
2.39	H 13, 14	

### 3.4 Anticancer effect of imp-A

**FIGURE 6:**
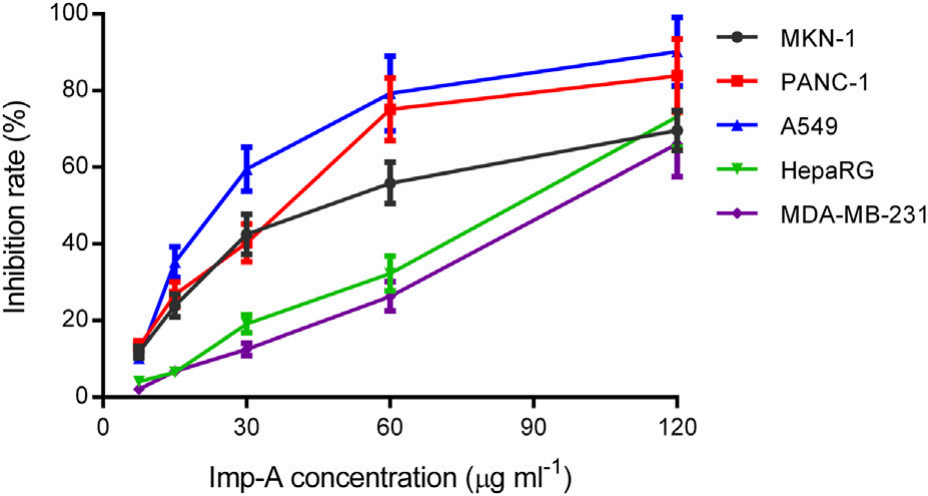
Inhibition rates of MKN-1, PANC-1, A549, HepaRG and MDA-MB-231 cells treated with imp-A at different concentrations.

A methyl thiazolyl tetrazolium (MTT) assay was performed to investigate the anticancer activities of imp-A. Mobocertinb is EGFR TKI and approved for the treatment of lung cancer. Imp-A was discovered during the forced degradation test of mobocertinib. As a result, A549 was chosen to evaluated the cytotoxicity of imp-A and mobocertinib. In addition, advanced gastric carcinoma, triple negative breast cancer, pancreatic carcinoma, hepatic carcinoma are easy to metastasis and have poor prognosis. Hence, MNK-1, MDA-MB-231, HepaRG and PANC-1 cells were also chosen. Figure 6 shows that imp-A inhibited the growth of tumour cells, including A549, MDA-MB-231, HepaRG, PANC-1 and MKN-1 cells. The corresponding IC50 values were 24.1, 90.4, 77.2, 33.2, and 47.0 μg mL^-1^. Half-maximal inhibitory concentration (IC50) indicates a drug can inhibit a biological process by half at this concentration, thus providing a measure of efficacy of a drug (Aykul and Martinez-Hackert, 2016). The lower IC50 value represents higher cytotoxicity (Gupta et al., 2024). Therefore, imp-A had the strongest anticancer effect on A549 cells. Imp-A exhibited relatively good activity in PANC-1 cells. Considering that limited drugs are available for treating pancreatic carcinoma, imp-A deserves to be further studied in the treatment of pancreatic carcinoma.

## 4 Conclusion

In this study, we have successfully developed a liquid-phase detection technique that can efficiently detect both the process impurities and degradation products of mobocertinib simultaneously which can provide convenience in process preparation studies and quality control. The detection technique exhibited good specificity, high sensitivity, and satisfactory linearity, precision, repeatability and robustness when validated according to ICH guidelines. It deserves to mention that a novel degradation product was discovered. We successfully revealed its structure and clarified its formation mechanism. Moreover, it shows satisfactory anticancer effects on various tumor cells. Future *in vivo* studies are to be explored to confirm the therapeutic effect of imp-A and its potential toxicity profile. We hope this novel product could be a promising therapeutic drug for cancer patients.

## Data Availability

The original contributions presented in the study are included in the article/Supplementary Material, further inquiries can be directed to the corresponding authors.
