# Supplementary material for: Reversed phase HPLC analysis of mobocertinib and its impurities and studies on the structure and biological activity of a new degradation product
Source: Front Chem. 2025 Sep 22;13:1659507. doi: 10.3389/fchem.2025.1659507 (PMC12497807; doi:10.3389/fchem.2025.1659507)
Supplement: Supplementary file 1 [file DataSheet1.pdf]

Supplementary Material

1 Supplementary Methods

1.1 Cell Culture

A549, MDA-MB-231, PANC-1 and MKN-1 cells were cultured in DMEM with 5% CO_2, 10% foetal bovine serum, 100 U mL^{-1} penicillin, and 100 μg mL^{-1} streptomycin at 37 °C. HepaRG cells were cultured in RPMI-1640 medium under the same conditions.

2 Supplementary results and discussion

2.1 Choice of solvent

In this study, mobocertinib had poor solubility in ACN but could be completely dissolved in MeOH or MeOH/H_2O (v/v, 1:1). Mobocertinib was stable in MeOH or MeOH/H_2O (v:v, 1:1), with no noticeable changes occurring at 12, 24, 48, or 72 h. Finally, considering solubility, stability, and cost, MeOH/H_2O (v:v, 1:1) was selected as the solvent in this study.

2.2 Optimization of HPLC conditions

The detection wavelength, mobile phase composition and elution gradient were evaluated to optimize the HPLC conditions. To determine the maximum absorption wavelength, UV－Vis spectra of mobocertinib and its impurities (1 mg mL^{-1}) were obtained from 200 nm to 400 nm. Figure S1 shows the recorded ultraviolet spectra. All seven substances exhibited a relatively strong absorption near 330 nm. As a result, 330 nm was chosen as the detection wavelength. A system suitability solution was used to determine the mobile-phase composition and elution gradient because of the high number and concentration of impurities and the presence of key peak pairs, which are difficult to separate. Different proportions of ACN-H_2O (90:10, 60:40) were detected, and chromatograms were recorded. Mobocertinib and its impurities had short retention times, and the resolution of adjacent peaks was poor in 90:10 ACN-H_2O, which did not satisfy the requirements (Figure S2A). Reducing the ACN proportion to 60% caused the peak width to increase and the column efficiency to decrease (Figure S2B). Hence, ACN was selected as the organic phase. ACN-H_2O (60:40) was used for isocratic elution. The retention times of the primary and impurity peaks became longer. However, the resolution of these peaks did not improve. Thus, gradient elution was applied and triethylamine was added to improve the peak profile. After several trials, the optimal mobile phase A was determined to be a mixture of an aqueous solution (pH 2.5) and ACN (9:1, v:v). The aqueous solution consisted of 0.025 mM KH_2PO_4 solution and 0.4% triethylamine. ACN was chosen as the mobile phase B. Several elution steps were tested to determine the optimal elution gradient. Unfortunately, the minimum resolution was 0.96 (<1.2) between adjacent impurity peaks and 1.01 (<1.5) between the primary peak and impurity peak under Condition 1 (0~2 min, at 10% solvent B; 2~20 min, from 10 to 80% solvent B; 20~30 min, at 80% solvent B; 30~31 min, from 80 to 10% solvent B; and 31~40 min, at 10% solvent B) (Figure S2C), which did not meet the requirements. Then, the rate of change of the gradient decreased and was set as follows: 0~2 min, at 10% solvent B; 2~30 min, from 10 to 80% solvent B; 30~40 min, at 80% solvent B; 40~41 min, from 80 to 10% solvent B; 41~50 min, at 10% solvent B (condition 2). More impurity peaks were recorded under Condition 2 than under

Condition 1. However, the resolutions were unsatisfactory (Figure S2D). Next, HPLC was performed under Condition 3. The results remained unsatisfactory (Figure S2E). The elution gradient was determined after several adjustments and is presented in Section 2.3. Under these conditions, the minimum resolutions for the mobocertinib peak and the impurity peaks were 1.68 and 2.48, respectively. Both resolutions were greater than 1.5. The minimum resolution was 1.81 between adjacent known impurity peaks and 1.42 between unknown impurity peaks, meeting the requirement (Figure 2A).

3 Supplementary Figures and Tables

3.1 Supplementary Figures

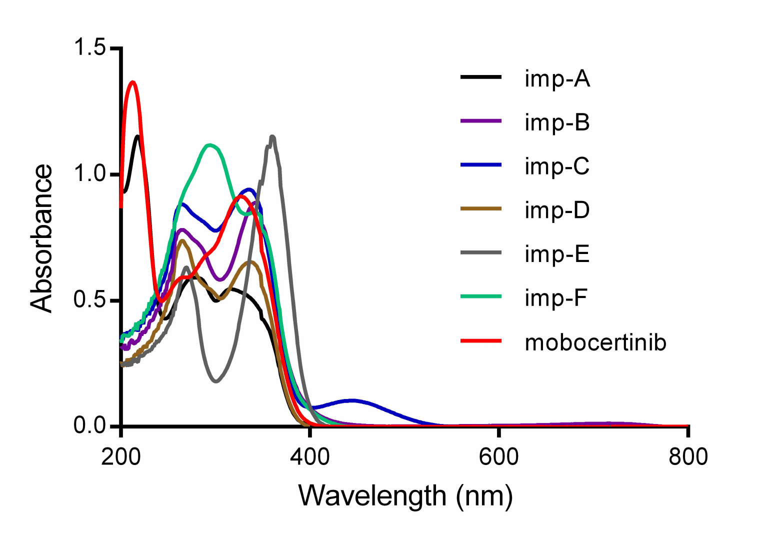

Figure S1. Ultraviolet spectra of mobocertinib and its impurities.

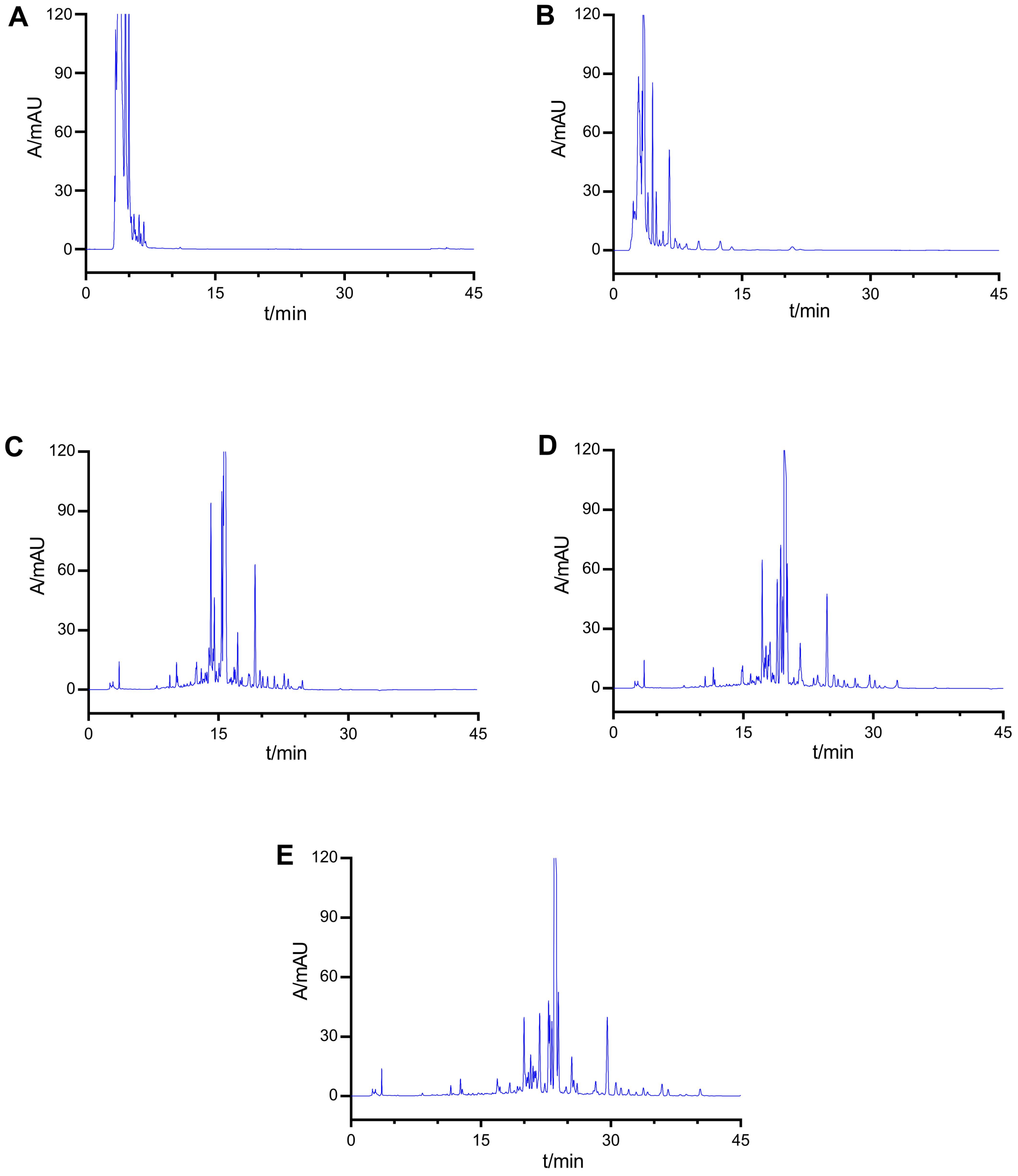

Figure S2. Chromatogram obtained under optimized liquid chromatography conditions: acetonitrile–water ratios of (A) 90：10 and (B) 60：40 and gradient elution under (C) Condition 1; (D) Condition 2; (E) Condition 3.

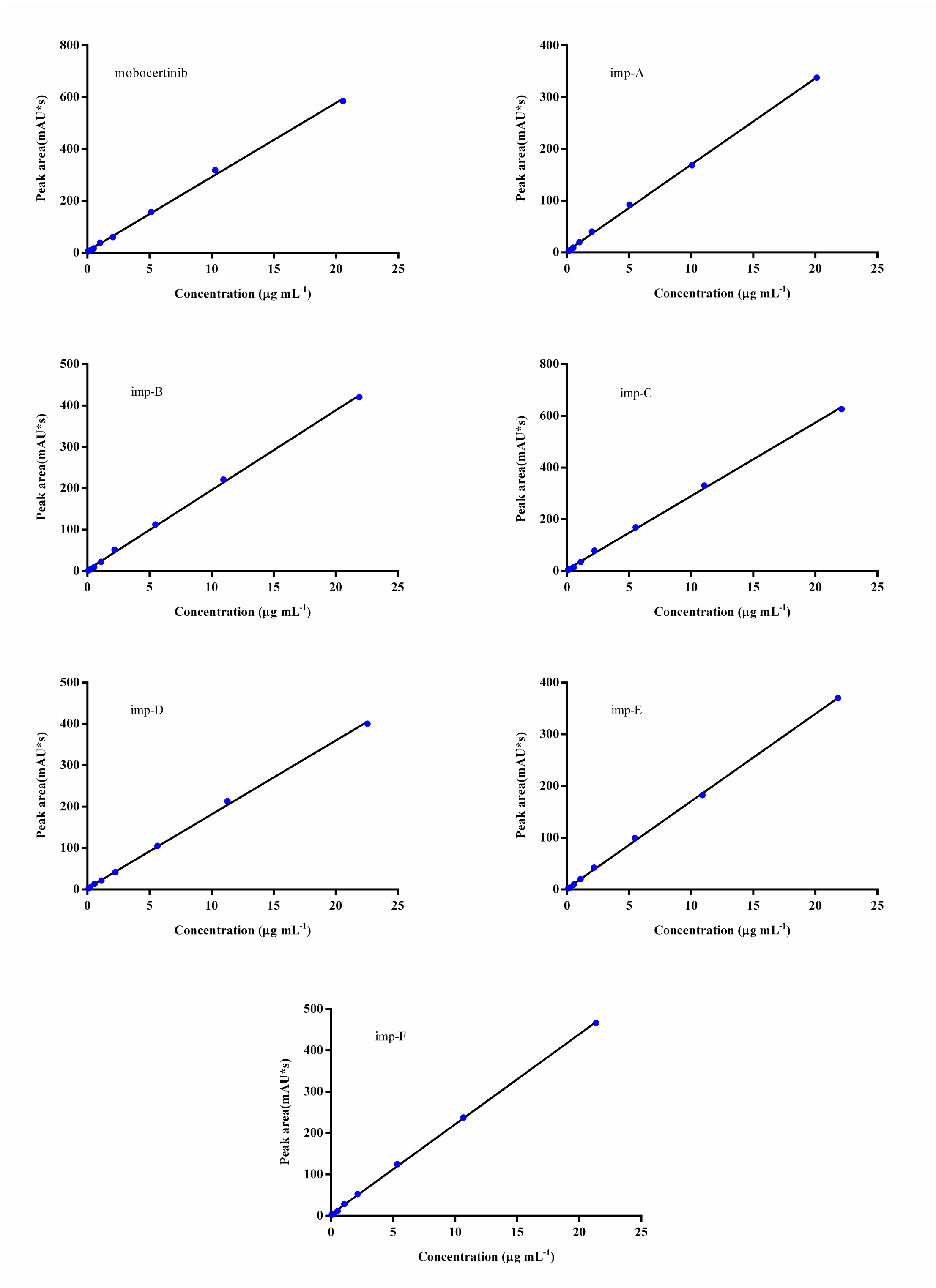

Figure S3. Regression curves of mobocertinib and its impurities.

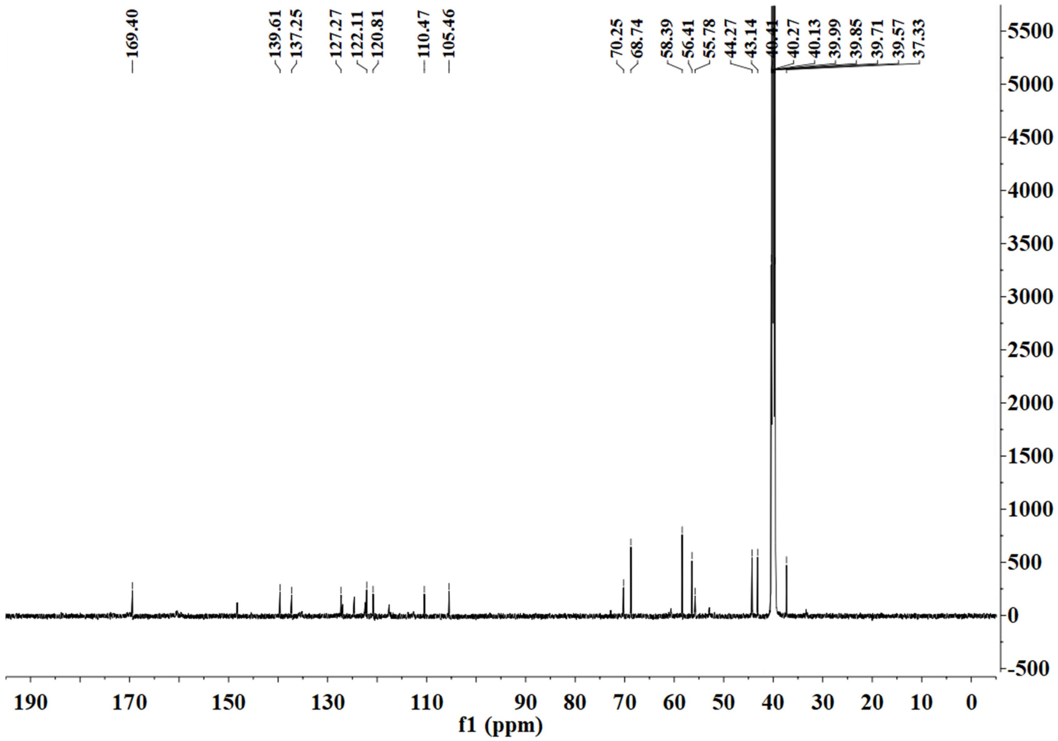

Figure S4. ^{13}C NMR spectrum of imp-A.

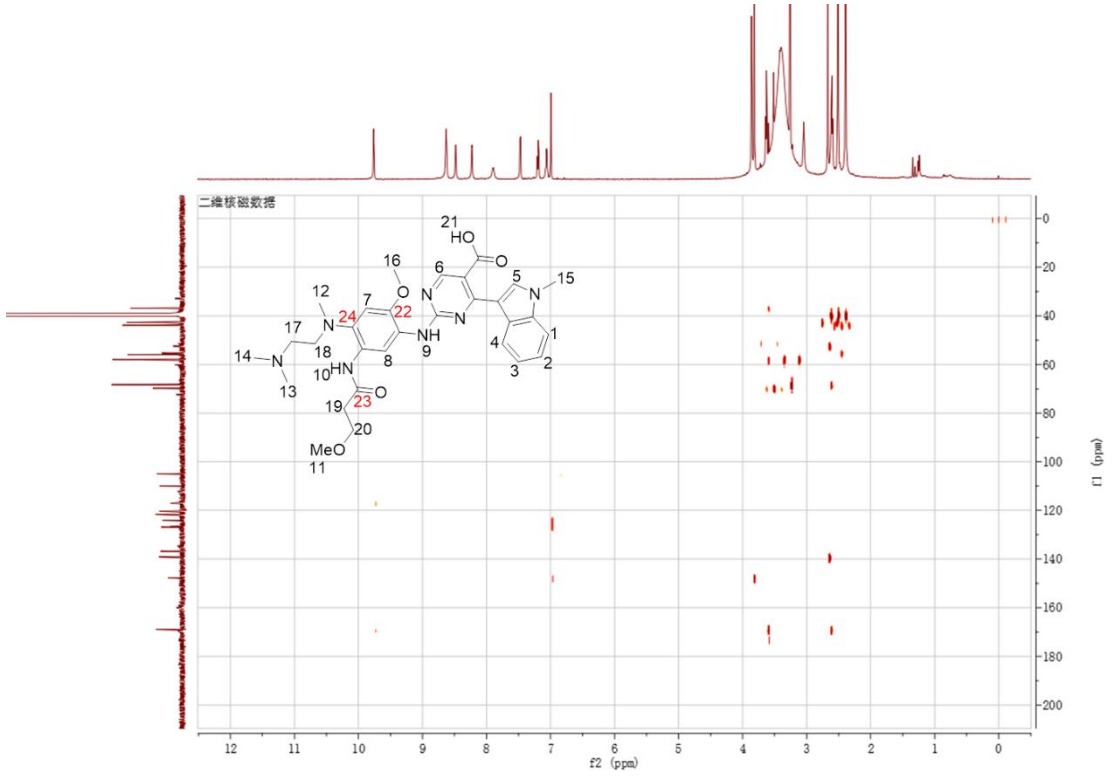

Figure S5. Full HMBC spectrum of imp-A.

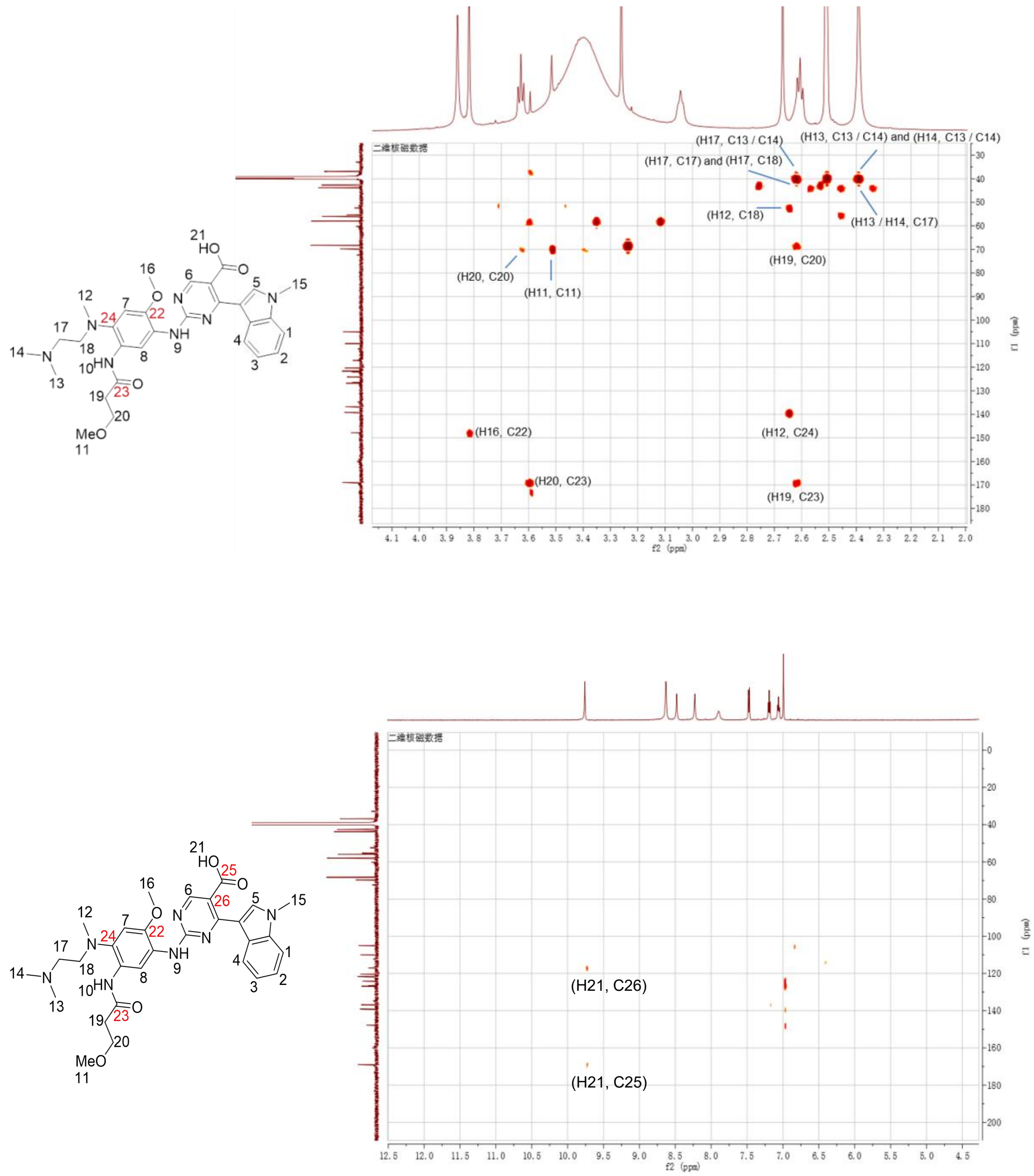

Figure S6. Locally amplified HMBC spectrum of imp-A.

3.2 Supplementary Tables

Table S1 Results of forced degradation tests.

Forced Degradation Condition	Number of impurities （>0.03 %）	Content of main peak (%)	Minimum resolution between main peak and impurities	Minimum resolution among impurities	Equilibrium (%)
Undegradation	4	99.43	5.45	3.01	100
Acid degradation	13	87.18	1.64	3.03	98.5
Base degradation	10	90.62	1.61	2.35	96.9
Oxidative degradation	12	92.38	2.49	1.72	95.3
Heat degradation	6	98.78	6.25	2.99	103.8
Photolytic degradation	12	88.23	2.44	1.59	102.3

Table S2 Recovery of impurities of mobocertinib.

substance	Concentration	Content in sample	Addition amount (μg)	Measured amount (μg)	Recovery rate (%)	Average recovery rate (%)	RSD (%)
imp-A	50%	0	1.027	0.959	93.38	95.64	2.11
		0	1.016	0.971	95.57		
		0	1.006	0.949	94.33		
	100%	0	2.054	1.953	95.08		
		0	2.032	1.928	94.88		
		0	2.012	1.892	94.04		

	150%	0	3.081	2.989	97.01		
		0	3.048	3.051	100.10		
		0	3.018	2.909	96.39		
imp-B	50%	0	1.012	0.935	92.39	97.68	3.84
		0	1.023	0.977	95.50		
		0	1.034	0.978	94.58		
	100%	0	2.024	2.102	103.85		
		0	2.046	2.105	102.88		
		0	2.068	2.029	98.11		
	150%	0	3.036	3.005	98.98		
		0	3.069	2.963	96.55		
		0	3.102	2.985	96.23		
imp-C	50%	0.17	1.025	1.142	94.83	97.58	3.21
		0.17	1.017	1.153	96.66		
		0.17	1.028	1.182	98.44		
	100%	0.17	2.050	2.214	99.71		
		0.17	2.034	2.212	100.39		
		0.17	2.056	2.271	102.19		
	150%	0.17	3.075	3.165	97.40		
		0.17	3.051	2.966	91.64		
		0.17	3.084	3.159	96.92		
imp-D	50%	0.13	1.017	1.085	93.90	93.97	2.34

		0.13	1.023	1.078	92.67		
		0.13	1.036	1.086	92.28		
	100%	0.13	2.034	2.151	99.36		
		0.13	2.046	2.065	94.57		
		0.13	2.072	2.065	93.39		
	150%	0.13	3.051	2.956	92.63		
		0.13	3.069	3.029	94.46		
		0.13	3.108	3.005	92.50		
imp-E	50%	0	1.022	0.966	94.52	96.72	3.11
		0	1.015	0.953	93.89		
		0	1.026	0.986	96.10		
	100%	0	2.044	1.985	97.11		
		0	2.030	1.956	96.35		
		0	2.052	1.902	92.69		
	150%	0	3.066	3.082	100.52		
		0	3.045	3.106	102.00		
		0	3.078	2.995	97.30		
imp-F	50%	0	1.017	1.007	99.02	98.45	3.78
		0	1.021	0.986	96.57		
		0	1.029	0.963	93.59		
	100%	0	2.034	2.053	100.93		
		0	2.042	2.115	103.57		
		0	2.058	2.062	100.19		

	150%	0	3.051	2.825	92.59		
		0	3.063	3.125	102.02		
		0	3.087	3.012	97.57		

Table S3 Test conditions for robustness.

Chromatogram conditions	The variation range of parameters
The initial proportion of mobile phases A-B (%)	92:8, 90:10, 88:12
Wavelength (nm)	325, 330, 335
Column temperature (℃)	35, 40, 45
Flow rate (mL min^{-1})	0.9, 1.0, 1.1
Mobile phase pH	2.4, 2.5, 2.6
Chromatographic column	Waters-C_{18}, Agilent-5HC-C_{18}, Gemini-C_{18}
